# Mania and hypomania associated with COVID-19: a series of 15 cases seen by the consultation-liaison psychiatry service in Qatar

**DOI:** 10.5339/qmj.2021.65

**Published:** 2021-11-30

**Authors:** Yousaf Iqbal, Majid Alabdulla, Javed Latoo, Rajeev Kumar, Sultan Albrahim, Ovais Wadoo, Peter M Haddad

**Affiliations:** ^1^Psychiatry Hospital, Hamad Medical Corporation, Doha, Qatar E-mail: yiqbal@hamad.qa; ^2^College of Medicine, Qatar University, Qatar; ^3^Division of Psychology and Mental Health, University of Manchester, UK

**Keywords:** COVID-19, SARS-Cov2, consultation-liaison psychiatry, mania/hypomania

## Abstract

Background: A range of neuropsychiatric diagnoses have been reported in association with coronavirus disease 2019 (COVID-19). However, only sporadic cases of mania or hypomania have been reported in patients with COVID-19. This study aimed to report clinical characteristics of 15 consecutive cases of COVID-19-associated mania or hypomania seen in three general hospitals in Qatar in the early months of the pandemic in 2020.

Methods: This study is a retrospective case-note review of 15 cases of COVID-19-associated mania or hypomania (confirmed by polymerase chain reaction test), seen as inpatient consultations out of the first 100 consecutive patients managed by consultation-liaison psychiatric teams in Qatar between 2 March 2020 and 7 July 2020.

Results: The mean age of the 15 patients was 40 years. Twelve patients had mania, and three had hypomania. Regarding the physical severity of COVID-19, 10 patients were asymptomatic, two had upper respiratory tract symptoms alone and three had pneumonia. None of the patients were intubated. Potential risk factors for mania/hypomania included pandemic-related psychosocial stress before admission (n = 9), past history of mania/bipolar disorder (n = 6) or psychosis (n = 2), raised inflammatory markers (n = 7) and steroid use (n = 3). None had a history of recent substance misuse. Other than one patient with advanced cancer, none had comorbidity regarded as likely to have caused mania or hypomania. Three patients had mild white matter ischaemic changes on brain imaging. Standard pharmacological treatment for mania (i.e. antipsychotic medication supplemented by prn benzodiazepines) was effective. Ten patients were discharged home from the COVID-19 facility where they presented, but five required transfer to Qatar's psychiatric hospital for further treatment of mania.

Conclusion: The association of mania or hypomania with COVID-19 may be spurious (e.g. representing an initial presentation of bipolar disorder) or causal. The reported cases illustrate a range of potential aetiological mechanisms by which COVID-19 could cause mania or hypomania. Cohort studies are necessary to determine the incidence, aetiology and prognosis of COVID-19-associated mania/hypomania.

## Introduction

Coronavirus disease 2019 (COVID-19) is caused by severe acute respiratory syndrome coronavirus 2 (SARS-CoV-2). COVID-19 primarily affects the respiratory system, but multiple organs and systems can be involved, and increasing attention has focused on the psychiatric effects.^1^ A health-record cohort study that investigated the incidence of neurological and psychiatric diagnoses of over 81 million patients, primarily across the USA found that nearly one-third of patients with COVID-19 received a neuropsychiatric diagnosis within 6 months of infection.^2^ These included intracranial haemorrhage, ischaemic stroke, encephalitis, dementia, psychosis, mood disorders, anxiety disorders, substance use disorder and insomnia. Although this study reported pooled incidence of all disorders and most individual disorders, which was higher than the control health conditions, it did not analyse cases of mania alone.

A few case reports have reported mania or hypomania in association with COVID-19.^3-6^ This could represent a spurious association, or alternatively, mania could be caused by COVID-19 through psychosocial and/or organic mechanisms. As per the Diagnostic and Statistical Manual of Mental Disorders, fifth edition (DSM-5), the patient must present with persistently elevated, expansive or irritable mood for at least 1 week or 4 consecutive days along with  ≥ 3 associated symptoms to qualify for the diagnosis of mania and hypomania, respectively. The associated symptoms include inflated self-esteem or grandiosity, decreased need for sleep, increased talkativeness, racing thoughts, distractibility, increased goal-directed activity or psychomotor agitation and engaging in risky activities.^7^ The International Classification of Diseases, 10th revision (ICD-10) similarly describes mania and hypomania as characterised by a persistent or mild elevation of mood with other associated symptoms leading to severe or mild disruption of work or social life, respectively.^8^ Mania can present with delusions (usually grandiose) or hallucinations. DSM-5 and ICD-10 allow the diagnosis of a manic episode caused by a medical disorder and medication or substance use.^7,8^ Medical disorders have been reported to cause mania, including thyroid abnormalities, various neurological conditions, and systemic inflammatory disorders among several others.^9-11^ Medications associated with mania include corticosteroids and sympathomimetics.^10^

Qatar is a peninsula in the Arabian Gulf, with a population of approximately 2.9 million, of which approximately 75% were men, and foreign nationals in Qatar make up more than 85% of the population.^12,13^ Herein, 15 consecutive cases of mania and hypomania associated with COVID-19 encountered in three general hospitals in Qatar from 2 March 2020 to 7 July 2020 are reported. During this period, Qatar recorded 100,936 confirmed COVID-19 cases.

## Methods

Qatar operates a publicly funded healthcare system. All residents, irrespective of nationality, are entitled to free or highly subsidised healthcare, including inpatient treatment. Consultation-liaison (CL) psychiatry services have been operating across all major hospitals, including the country's main COVID-19-designated hospital. All psychiatric assessments within the CL service are conducted by consultant psychiatrists or psychiatry fellows under the supervision of a consultant psychiatrist.

The CL services conducted a retrospective case-note review of the first 100 consecutive referrals of patients who tested positive for SARS-CoV-2 since the start of the pandemic to offer a good representation of patients with various psychiatric presentations in this setting. The clinical characteristics of the first 50 of such referrals have been reported,^5^ including the data extraction details from the clinical records. The inclusion criteria were patients aged  ≥ 18 years who had a positive antigen test (real-time polymerase chain reaction test for SARS-CoV-2) during the index hospitalisation. From each clinical record, patients’ sociodemographic and clinical data were retrieved using a standardised data abstraction form. Data were abstracted for the whole hospitalisation; in some cases, the CL team conducted several assessments during this period. Two research team members (Y.I. and S.A.) extracted the data with regular discussions to ensure consistency in data recording. Rating uncertainties were resolved by discussion with a third team member. Diagnoses reflected the psychiatric diagnosis made at discharge after the index admission (i.e. the admission that involved the CL referral). Diagnoses reflected clinical judgement and were not operationally defined. In this study, clinical details of all cases of mania and hypomania seen within an extended cohort of 100 patients were reported. Patients with mania and hypomania were referred to the CL team from 2 March to 7 July 2020. A diagnosis of mania, rather than hypomania, required the presence of psychotic symptoms and/or marked behavioural disturbance or functional impairment due to manic symptoms.

The study was approved by the Hamad Medical Corporation Institutional Review Board (MRC-05–072).

## Results

The consecutive series of 100 patients with COVID-19 included 15 cases of mania and hypomania. Table 1 summarises the demographic and clinical characteristics of those 15 cases. Six patients had a past history of bipolar affective disorder, of whom three had poor adherence to maintenance mood stabilisers or antipsychotic medication before admission. Another two patients had a past history of psychosis, and one had unipolar depression history. None of the patients had evidence of recent substance misuse. Of the 15 patients, 10 had no physical symptoms of COVID-19, two had mild upper respiratory tract symptoms and three had COVID-19 pneumonia, but none required intubation. These patients were admitted to the COVID-19 sites primarily because of COVID-19-associated mental health problems.

Steroids were prescribed to three patients. One patient had a pre-existing neuropsychiatric condition (well-controlled epilepsy). Only one patient had an organic disorder, other than COVID-19, that was regarded as a possible aetiological factor for the hypomanic or manic episode; this was a patient with brain metastases (primary lung cancer) and an existing diagnosis of bipolar disorder. The metastasis and/or recent cancer treatment might have been partly responsible for his manic relapse.

Table 2 summarises investigations, treatment and follow-up data, where available. Brain imaging (computed tomography, magnetic resonance imaging or both) was conducted in 12 patients; three (36, 50 and 52 years old) had mild white matter ischaemic changes, and another patient, already referred to, had multiple brain metastases. Seven patients had raised peripheral inflammatory markers.

All patients responded well to standard treatment for mania or hypomania. Upon discharge from COVID-19-designated hospitals, five patients were transferred to Doha's psychiatry hospital for the treatment of on-going manic symptoms, whilst the remaining 10 were discharged from the COVID-19-designated facilities to their homes.

Follow-up data of up to 6 months was available for 11 patients; six remained psychiatrically well, three experienced a manic relapse requiring hospitalisation and one had a depressive episode treated as an outpatient. Two of the four patients who relapsed had an existing psychiatric diagnosis before the index admission, with one of them having a bipolar diagnosis.

## Discussion

To the best of our knowledge, this is the largest case series of mania and hypomania associated with COVID-19. The coexistence of mania or hypomania and COVID-19 makes it reasonable to consider COVID-19 as a potential aetiological factor. However, this cannot be proved, and the association may be coincidental. A cohort study of patients with acute COVID-19, compared with a control acute medical event, would be necessary to investigate causality. Nevertheless, our case series builds up on the existing literature by suggesting some potential mechanisms by which COVID-19 infection, and the pandemic more broadly, could be a risk factor for mania or hypomania:

• Psychosocial stress related to the pandemic, most commonly social isolation and financial difficulties, was documented in nine cases. Life events are recognised to precede mania, though it is difficult to determine whether life events are a cause or effect of mood symptoms.^14^
• Raised peripheral inflammatory markers were reported in seven cases. Pro-inflammatory cytokines are elevated during mania,^15^ and COVID-19 is associated with an inflammatory state.^16^ Exactly how inflammation may cause mania is unclear, but potential mechanisms include disruption of the blood–brain barrier, glial cells and neuro-endocrine and neurotransmitter systems.^17^
• A past history of bipolar affective disorder was present in six cases, of which there was evidence of poor medication adherence in three cases. There was insufficient information to know whether non-adherence reflected the pandemic causing disruption of these patients’ psychiatric care or medication supply.
• Steroids were prescribed to three patients. Steroids can cause mania^10^ and have been implicated in some reports of COVID-19-associated mania.^18^

Three patients had mild white matter ischaemic changes. This may be a coincidental finding that predated COVID-19. Alternatively, the lesions could be due to COVID-19, with possible mechanisms including hypoxia, inflammation and a hypercoagulable state.^19,20^ Inflammation appears the most plausible in our cases. SARS-CoV-2 may also cause neuropsychiatric disorders by a direct neurotropic effect.^21^ Cerebrospinal fluid analysis was not performed in our cases, making it impossible to comment further on this.

Sleep disturbance was the most common psychiatric symptom documented in our case series. This could be a symptom of mania or hypomania, a direct effect of COVID-19 illness and/or hospitalisation. Insomnia can trigger mania and hypomania.^22^

In our series, one patient had concurrent delirium and mania. Manic delirium is a controversial diagnosis, but it is well documented in the literature.^23^ No other patients in our series had symptoms suggestive of delirium.

Treatment of several cases was challenging, as a disinhibited behaviour can disrupt infection control measures. The pharmacological treatment used was consistent with evidence-based mania guidelines and consisted of an antipsychotic with benzodiazepines to provide additional control of disturbed behaviour.^24^ Improvement in the manic or hypomanic illness was documented in all cases upon hospital discharge.

One of the challenges of the CL team was a diverse ethnic population speaking different languages. Where possible, members of the CL team who spoke the patients’ native language were allocated to conduct assessments.

## Limitations

Our case series is limited to patients with mania and hypomania during an index admission for COVID-19 at a COVID-19 facility or general hospital referred to the CL team. Given the behavioural disturbance associated with mania, it is reasonable to assume that all manic cases were referred. However, some hypomanic cases may have gone unreferred. This study also excluded manic and hypomanic cases that occurred soon after recovery from acute COVID-19; as these patients are not infectious, they would be assessed or treated at the main psychiatric hospital and not admitted to a COVID-19 facility or general hospital with subsequent referral to the CL team. Furthermore, as this was a retrospective case series, there may possibly be some elements of missing data; thus, further information about the socioeconomic status, education level and level of family support might have strengthened the paper.

## Conclusion

Mania or hypomania with COVID-19 may represent a spurious (e.g. an initial presentation of bipolar affective disorder) or causal association. In this study, psychosocial stress, increased levels of inflammatory markers and past history of bipolar affective disorder were potential risk factors for mania or hypomania. Sleep disturbance was the most common symptom. Outcomes of COVID-19-associated mania and hypomania were favourable with standard pharmacological treatment. CL psychiatry services play an important role in undertaking comprehensive assessment, establishing diagnoses and collaborative work with the medical team in offering on-going holistic care whilst following strict infection control measures. CL services also assist with providing psychoeducation to patients and offering support and reassurance regarding COVID-19. Cohort studies, with larger sample sizes, are necessary to determine the incidence, aetiology and longer-term prognosis of COVID-19-associated mania and hypomania.

## Declarations

### Ethical approval and consent to participate

The study was approved by the Institutional Review Board of Hamad Medical Corporation (MRC-05–072).

### Consent for publication

Not applicable

### Availability of data and materials

The data that support the findings of this study are available from the corresponding author upon reasonable request and pending additional ethical approval.

### Competing interests

PMH reports personal fees from Janssen, Lundbeck, Otsuka, NewBridge Pharmaceuticals and Sunovion, outside the submitted work.

### Funding

This study did not receive any specific grant from funding agencies in the public, commercial, or not-for-profit sectors.

### Authors’ contribution

All authors contributed to the design of the study, interpretation of the data and drafting and revising the manuscript. M.A., Y.I. and P.M.H. conceptualised the study. Y.I. and S.A. collected and analysed the data. P.M.H. and Y.I. conducted data analysis.

### Acknowledgements

None

## Figures and Tables

**Table 1:** Demographic and clinical characteristic of the 15 consecutive patients diagnosed with COVID-19-associated mania or hypomania

Characteristics	Value

Age (Mean and range), (years)	40 (23–66)

Sex (n)	

Male	14

Female	1

Ethnicity (n)	

Qatari	4

South Asian (Indian, Pakistani, Bengali and Nepali)	10

South-East Asian (Philippines)	1

Severity of COVID infection* (n)	

Asymptomatic	10

Mild COVID-19	2

Mild COVID-19 pneumonia	1

Severe COVID-19 pneumonia	2

Critical COVID-19	0

Past psychiatric history	

Bipolar affective disorder	6

Psychosis other than bipolar disorder	2

Unipolar depression	1

No past psychiatric history	6

Significant pandemic-related psychosocial stressor prior to admission (n)	

Present	9

Absent	6

Steroids prescribed (n)	

Yes	3

No	12

Pre-existing neuropsychiatric disorder	

Present	1**

Absent	14

CL diagnosis (n)	

Mania	12

Hypomania	3

Psychiatric symptoms	

Insomnia	13

Elation	10

Abnormal behaviour	10

Delusions	9

Irritability	9

Agitation	8

Aggressive behaviour	8

Anxiety	7

Impaired concentration,	5

Persecutory beliefs	5

Auditory hallucinations	5


*COVID-19 disease severity (Communicable Disease Centre Criteria as per the treatment protocol of Hamad Medical Corporation)

• Asymptomatic, i.e. no characteristic physical symptoms of COVID-19 infection

• Mild COVID-19: uncomplicated upper respiratory tract viral infection, may have non-specific symptoms such as fever, cough, sore throat, nasal congestion, malaise, headache, muscle pain or malaise. Older and immunosuppressed groups may present with atypical symptoms

• Mild pneumonia: patients with pneumonia and no signs of severe pneumonia

• Severe pneumonia: fever or suspected respiratory infection, plus one of the following:

– respiratory rate>30 breaths per min

– severe respiratory distress, or SpO2 < 90% on room air

• Critical disease: acute respiratory distress syndrome (ARDS), sepsis, septic shock

** One patient had epilepsy, which was well controlled

**Table 2:** Investigation, treatment and outcomes of the 15 consecutive patients diagnosed with COVID-19-associated mania or hypomania

Characteristics	Values

Brain scans (CT or CT and MRI)	Brain imaging was conducted in 12 patients with four showing abnormalities:

	1 case: metastases

	3 cases: mild white matter ischaemic changes

Raised inflammatory markers*, (n)	7 patients had raised inflammatory markers, i.e.

	- Increased CRP levels (n = 4) ranging from 38.2 to 366.8 mg/L

	- Increased neutrophil count (n = 3) ranging from 10.9 to 21.6 × 10^3^ /μL

Psychotropic medications prescribed, (n)	

Olanzapine	12

Benzodiazepine (lorazepam or clonazepam)	9

Haloperidol	6

Antihistamines (diphenhydramine/promethazine)	5

Valproate	4

Zolpidem	3

Quetiapine	2

Duration of inpatient stay in the COVID-19 facility (mean and range), (days)	21.33 (8–51)

Patients requiring transfer to psychiatric hospital	

Yes	5

No	10

Relapse during 6-month follow-up (for 11 patients where follow-up information was available) (n)	

Yes	4

No	11


*Raised inflammatory markers: The normal values of CRP level and neutrophil count are 0–5 mg/L and 2-7 × 10^3^ /μL, respectively
